# Kaposiform hemangioendothelioma associated with coagulopathy and treated with low-dose aspirin: a three-case series^؆^

**DOI:** 10.1016/j.abd.2026.501323

**Published:** 2026-03-26

**Authors:** Luciana Baptista Pereira, Guilhane Maria Magalhães, Daniel Dias Ribeiro, Marcelo de Mattos Garcia, Katherynne Bezerra Marques, João Renato Vianna Gontijo

**Affiliations:** a Internal Medicine Department, School of Medicine, Universidade Federal de Minas Gerais, Belo Horizonte, MG, Brazil; b Dermatology Service, Hospital das Clínicas, Universidade Federal de Minas Gerais, Belo Horizonte, MG, Brazil; c Hematology and Oncology Functional Unit, Hospital das Clínicas, Universidade Federal de Minas Gerais, Belo Horizonte, MG, Brazil; d Radiology Department, Clínica Axial, Belo Horizonte, MG, Brazil

Dear Editor,

Kaposiform hemangioendothelioma (KH) is a rare angiomatous tumor, potentially lethal due to coagulopathies. It is usually a single tumor and occurs in the neck, extremities, and trunk.^1^, ^2^ Approximately 93% of the tumors begin in childhood, being present at birth in 60% of cases.^1^, ^2^

The Kasabach-Merritt phenomenon (KMP) is a clinical-laboratory entity characterized by platelet capture in the vessels.^1^, ^3^ Thrombocytopenia (platelets <100,000 mL), hypofibrinogenemia (< 160 mg/L), and increased D-dimer (> 2 times the reference value) are present.^3^

KH and tufted angioma are entities of the same spectrum, sharing clinical, histopathological, and molecular characteristics. Three patterns can be distinguished: without complications; with KMP; with chronic coagulopathy, but without thrombocytopenia.^1^, ^2^, ^3^, ^4^, ^5^ This case report describes the long-term follow-up of three children with histopathologically confirmed KH and coagulopathies treated with Acetylsalicylic acid (ASA).

## Case 1

**Figure 1:**
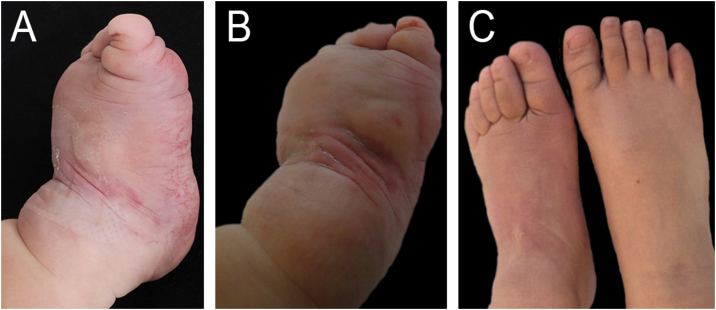
Patient 1 (A) Infiltrative angiomatous tumor affecting mainly the anterior and middle part of the foot, at four months of age. (B) At ten months of age, after five months of systemic corticosteroids – lesion with hardened consistency, without significant decrease in volume. (C) At six years of age – after using ASA, the feet acquired a normal diameter, allowing the child to wear shoes of the same size.

**Figure 2:**
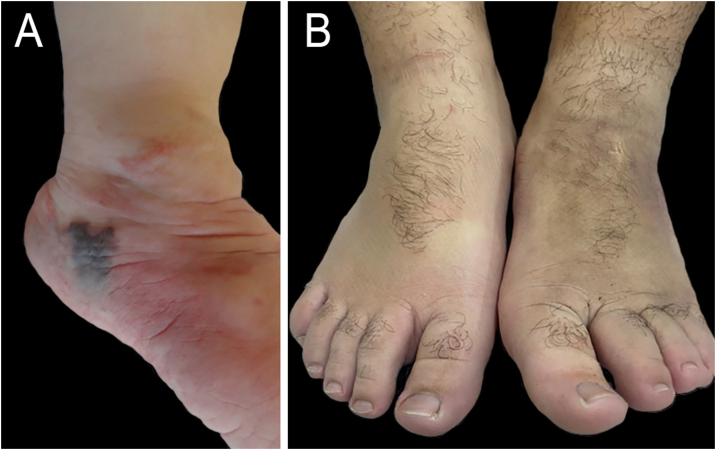
Patient 1(A) Appearance at three years of age after an attempt to discontinue aspirin – appearance of ecchymosis and increased volume. (B) At 16 years of age, maintaining aspirin use at a dose of 100 mg/day.

A male child presented with an angiomatous tumor on the left foot, present at birth and with progressive growth (Fig. 1). Platelets and fibrinogen levels were normal. Prednisolone (2 mg/kg/day) was started at five months of age and discontinued at 12 months of age without improvement. The lesion continued to increase in size, with ecchymosis, suggesting KH with chronic coagulopathy, without thrombocytopenia. At two years of age, aspirin 200 mg (10 mg/kg/day) was introduced, resulting in a decrease in tumor size and subsequent dose reduction to 100 mg/day. This dose has been maintained to date (15 years of follow-up; Fig. 2). Attempts to discontinue the medication result in an increase in lesion size and the appearance of ecchymosis.

## Case 2

**Figure 3:**
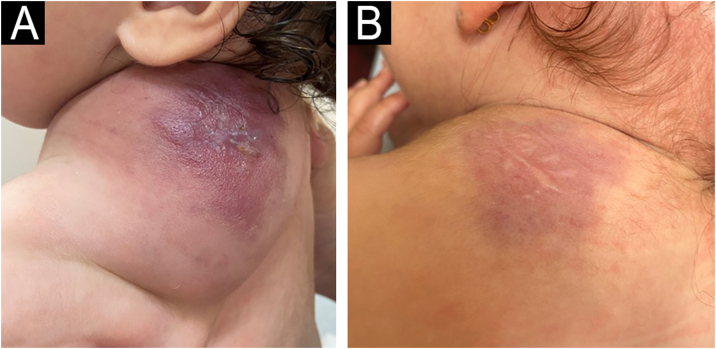
Patient 2 (A) Angiomatous tumor in the posterior region of the left trunk (trapezius region), at four months of age, before treatment with ASA and while using oral prednisolone. (B) Appearance at 18 months of age, without oral corticosteroids and maintaining ASA use.

A four-month-old female infant presented with an angiomatous macule noted at 40 days of age on the posterior trunk, showing progressive growth (Fig. 3). Prednisolone (1 mg/kg/day) was started at three months, without improvement: Hb was 10.4 g/dL; platelets 20,000 mL; fibrinogen 72 mg/L; D-dimer 23,230 ng/mL. At four months, 50 mg of aspirin (7 mg/kg/day) was introduced, with laboratory improvement within one month (Hb 13.4 g/dL; platelets 67,000 mL; D-dimer 2,252 ng/mL) and a decrease in volume. Corticosteroid dose was tapered and discontinued at five months. At 12 months, the tests returned to normal. The last evaluation was at 18 months showed normal tests and aspirin was maintained.

## Case 3

**Figure 4:**
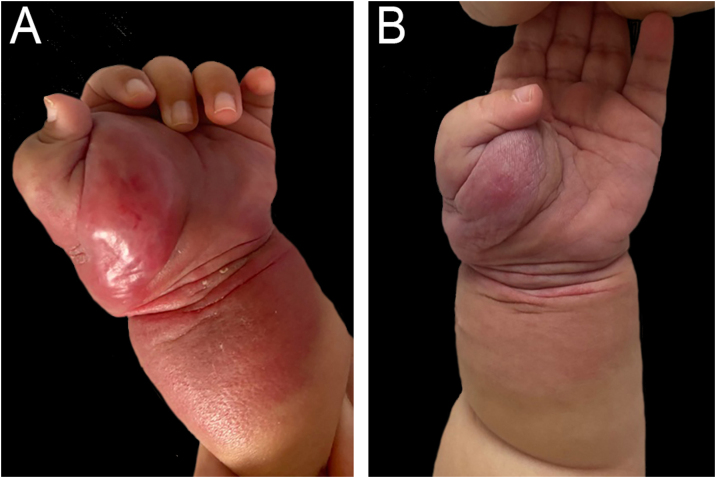
Patient 3 (A) Angiomatous tumor in the left upper limb, at three months of age, before the start of treatment with ASA and while using oral prednisolone. (B) At five months, with a significant decrease in volume, two months after starting ASA.

A female infant presented with an angiomatous tumor at birth on her left upper limb (Fig. 4) and thrombocytopenia (89,000 mL). The lesion worsened, and she developed anemia (Hb 10.4 g/dL), hypofibrinogenemia (118 mg/L), and worsening thrombocytopenia (14,000 mL). Prednisolone (1 mg/kg/day) was initiated, resulting in a slight improvement in volume and platelets (37,000 mL). At three months of age, aspirin 50 mg/day (10 mg/kg/day) was introduced. There was a significant decrease in volume and improvement in laboratory results (Hb 15.1 g/dL; platelets 398,000 mL; fibrinogen 190 mg/L), and prednisolone was discontinued. The last evaluation was at five months, with corticosteroid being discontinued and aspirin maintained.

KH treatment aims to reduce tumor size and correct coagulopathy, but may not be curative regardless of the medication used. Surgical treatment is considered the gold standard, but resection is usually not feasible. Oral corticosteroids have been considered first-line treatment but are often ineffective when used alone. Vincristine can be used (70% efficacy) but requires central access for its use.^2^ Sirolimus is considered the first-line therapeutic option with a 91% efficacy rate.^2^, ^3^ Antiplatelet agents such as ASA and ticlopidine have been used alone or in combination with other drugs.^5^, ^6^, ^7^, ^8^, ^9^

In 1992, ASA (10 mg/kg/day) with ticlopidine (10 mg/kg/day) were used to treat KMP in a three-month-old infant with no prior treatments. The authors suggested considering them as first-line drugs, as they potentially have fewer side effects compared to other medications.^6^

One concern with prolonged ASA use would be Reye's syndrome, but it is considered low risk when used at low doses. ASA should be discontinued for vaccinations and during viral infections.^7^ The incidence rate of intracranial bleeding associated with the use of low doses of ASA in adults is approximately 0.6–1.4/1000 patients/year and for gastrointestinal bleeding, 3.5–3.7/1000 patients/year. Data on the incidence of bleeding associated with ASA in children are extremely weak.^10^

In Brazil, sirolimus is not available for the treatment of vascular tumors in the Brazilian Public Health System (SUS), and vincristine requires special care for its use. ASA is an inexpensive, safe and effective option, and can be considered the first choice for the treatment of KH in developing countries. It is worth noting that its continued use may be necessary.

## ORCID ID

Luciana Baptista Pereira: 0000-0002-8548-9938

Guilhane Maria Magalhães: 0000-0003-4839-0098

Daniel Dias Ribeiro: 0000-0002-5257-9507

Marcelo de Mattos Garcia: 0000-0003-4518-5814

Katherynne Bezerra Marques: 0009-0006-9994-7991

## Research data availability

Does not apply.

## Financial support

None declared.

## Authors' contributions

Luciana Baptista Pereira: Drafting and editing of the manuscript; critical review of important intellectual content; approval of the final version of the manuscript.

Guilhane Maria Magalhães: Drafting and editing of the manuscript; critical review of important intellectual content; approval of the final version of the manuscript.

Daniel Dias Ribeiro: Drafting and editing of the manuscript; critical review of important intellectual content; approval of the final version of the manuscript.

Marcelo de Mattos Garcia: Drafting and editing of the manuscript; critical review of important intellectual content; approval of the final version of the manuscript.

Katherynne Bezerra Marques: Drafting and editing of the manuscript; critical review of important intellectual content; approval of the final version of the manuscript.

João Renato Vianna Gontijo: Drafting and editing of the manuscript; critical review of important intellectual content; approval of the final version of the manuscript.

## Conflicts of interest

None declared.
